# Supplementary material for: Impact of Health Access on Hospitalization of Children and Youth with Cerebral Palsy and Paralytic Syndromes in Brazilian Regions
Source: Int J Environ Res Public Health. 2024 Sep 26;21(10):1286. doi: 10.3390/ijerph21101286 (PMC11507291; doi:10.3390/ijerph21101286)
Supplement: Supplementary file 1 [file ijerph-21-01286-s001.zip › Figure 1 Uneven vaccination.pptx]

## Slide 1
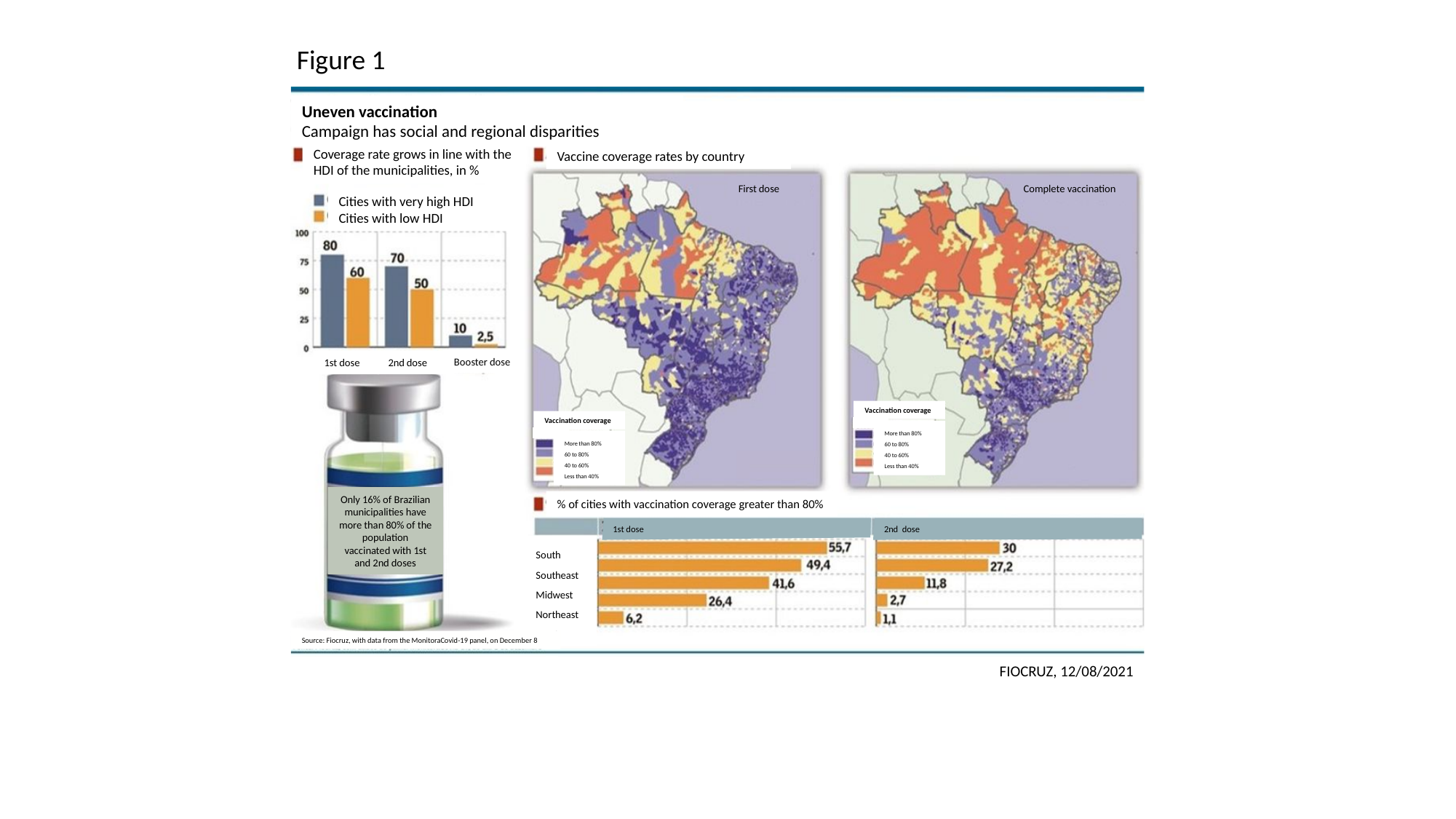

Figure 1
Figura 1
FIOCRUZ, 12/08/2021
Uneven vaccination
Campaign has social and regional disparities
Coverage rate grows in line with the HDI of the municipalities, in %
Vaccine coverage rates by country
First dose
Complete vaccination
Cities with very high HDI
Cities with low HDI
Booster dose
1st dose
2nd dose
Vaccination coverage
Vaccination coverage
More than 80%
60 to 80%
40 to 60%
Less than 40%
More than 80%
60 to 80%
40 to 60%
Less than 40%
Only 16% of Brazilian municipalities have more than 80% of the population vaccinated with 1st and 2nd doses
% of cities with vaccination coverage greater than 80%
2nd dose
1st dose
South
Southeast
Midwest
Northeast
North
Source: Fiocruz, with data from the MonitoraCovid-19 panel, on December 8
