# Supplementary material for: Impact of Health Access on Hospitalization of Children and Youth with Cerebral Palsy and Paralytic Syndromes in Brazilian Regions
Source: Int J Environ Res Public Health. 2024 Sep 26;21(10):1286. doi: 10.3390/ijerph21101286 (PMC11507291; doi:10.3390/ijerph21101286)
Supplement: Supplementary file 1 [file ijerph-21-01286-s001.zip › Chart 1 Health Care Facilities and Physicians working in Health Care Facilities (DATASUS).pptx]

## Slide 1
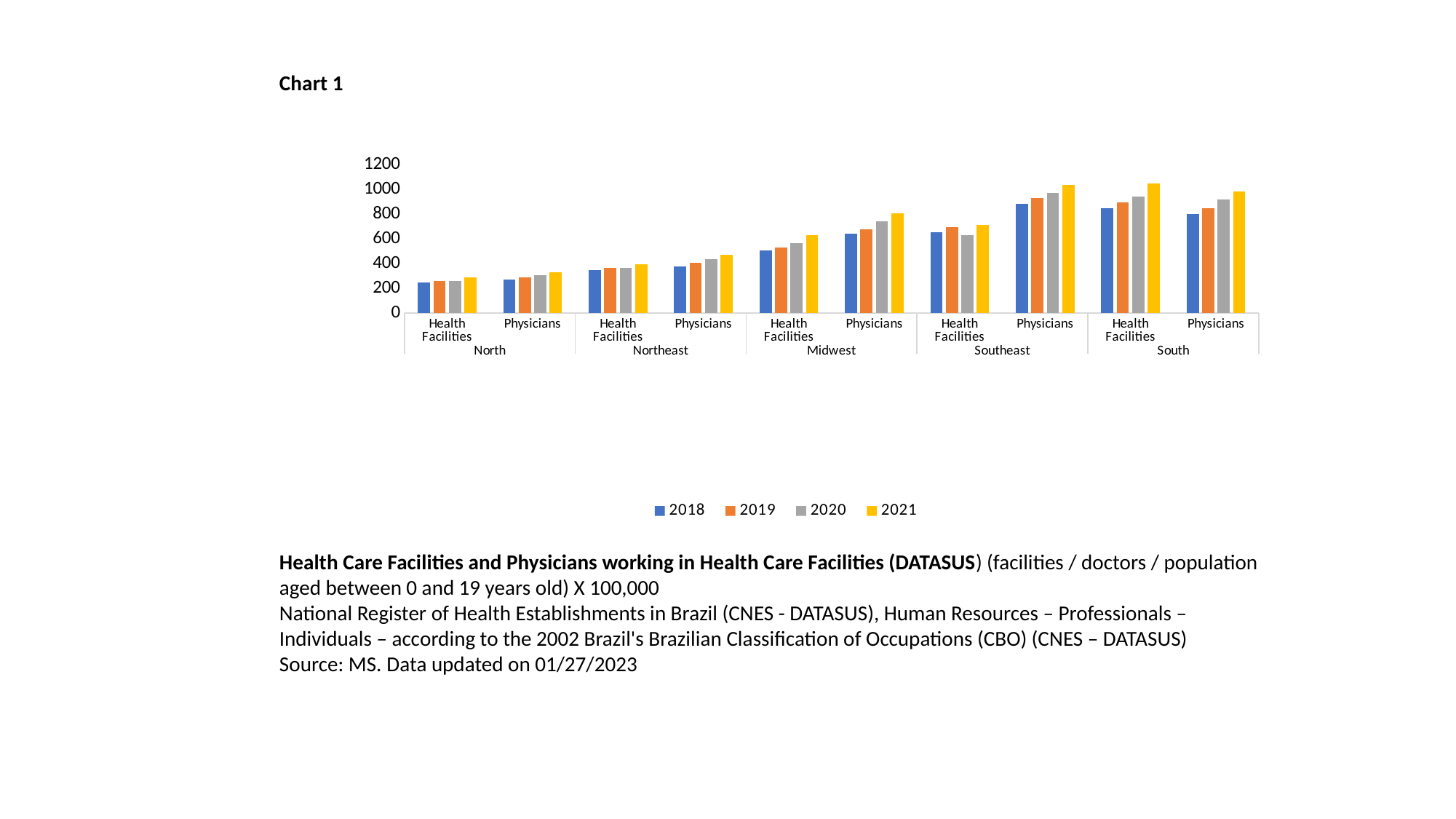

Chart 1
Health Care Facilities and Physicians working in Health Care Facilities (DATASUS) (facilities / doctors / population aged between 0 and 19 years old) X 100,000
National Register of Health Establishments in Brazil (CNES - DATASUS), Human Resources – Professionals – Individuals – according to the 2002 Brazil's Brazilian Classification of Occupations (CBO) (CNES – DATASUS)
Source: MS. Data updated on 01/27/2023
### Chart
| Category | 2018 | 2019 | 2020 | 2021 |
|---|---|---|---|---|
| Health Facilities | 248.1689890618618 | 257.536553836779 | 256.4247127963098 | 287.27591070822945 |
| Physicians | 269.7107246379357 | 285.24972647791066 | 305.8016406552532 | 327.01846643169677 |
| Health Facilities | 348.0969835984685 | 362.9763390150218 | 363.44257645553654 | 395.0637140420355 |
| Physicians | 378.5868856482809 | 404.2022707798068 | 432.31893791691385 | 470.42752661065805 |
| Health Facilities | 504.3566143861927 | 531.2191916213453 | 563.8526904623395 | 630.310606280172 |
| Physicians | 641.9121730164359 | 677.823238059033 | 739.7283744088583 | 803.3246931299672 |
| Health Facilities | 651.6748441058459 | 691.6787275173156 | 631.8473881488542 | 708.6417670328171 |
| Physicians | 883.5261516284965 | 927.2395587735969 | 969.0556968829443 | 1035.4500076266181 |
| Health Facilities | 845.7375040902251 | 892.3227665097559 | 938.815496194821 | 1048.1855156845254 |
| Physicians | 797.108063285331 | 847.6928030759107 | 917.9181855032916 | 980.1854255158133 |
